# Resveratrol-4-*O*-d-(2'-galloyl)-glucopyranoside Isolated from *Polygonum cuspidatum* Exhibits Anti-Hepatocellular Carcinoma Viability by Inducing Apoptosis via the JNK and ERK Pathway

**DOI:** 10.3390/molecules19021592

**Published:** 2014-01-27

**Authors:** Qichao Xie, Yupeng Yang, Zhiyi Wang, Fanglin Chen, Anmei Zhang, Chengcheng Liu

**Affiliations:** Department of Oncology, the Second Affiliated Hospital, Third Military Medical University, Chongqing 400037, China; E-Mails: yangypxqh@126.com (Y.Y.); wangzyxqh@126.com (Z.W.); chenfanglin2012@163.com (F.C.); amayzhang@yeah.net (A.Z.); liu_xqh@163.com (C.L.)

**Keywords:** resveratrol-4-*O*-D-(2'-galloyl)-glucopyranoside, hepatocellular carcinoma, apoptosis, JNK, ERK

## Abstract

Resveratrol-4-*O*-d-(2'-galloyl)-glucopyranoside (RESG) is one of the active compounds isolated from *Polygonum cuspidatum*. The purpose of our present study was to investigate the anti-hepatocellular carcinoma effect of RESG *in vitro* and *in vivo*, and the possible mechanisms *in vitro*. *In vitro*, our results showed that RESG could significantly inhibit the human hepatocellular carcinoma viability in the MTT assay, in a dose- and time-dependent manner. Furthermore, our results demonstrated that RESG could induce SMMC-7721 cell apoptosis and activate caspases 3 and caspases 9 by using Annexin V-FITC staining and western blot, respectively. *In vivo*, RESG also showed efficacy in SMMC-7721 xenograft model in nude mice, and further molecule mechanisms were investigated *in vitro*. The results showed that RESG up-regulated the p-JNK expressions, whereas it down-regulated the p-ERK expressions. Above results demonstrated that RESG is a potential therapeutic agent for hepatocellular carcinoma via JNK and ERK pathway to induce apoptosis. Our finding provided a basis for further development of RESG as an anticancer agent.

## 1. Introduction

*Polygonum cuspidatum* (Polygonaceae), a traditional Chinese medicinal herb and named *Huzhang* in Chinese, is a large herbaceous perennial plant, widely distributed in southern China, Japan and Korea [1,2]. The functions of *P. cuspidatum* root in Chinese Traditional Medicine theory system are activating blood circulation, removing blood and releasing pain, expelling wind and dampness, and clearing away heat, harmful and toxic materials [3]. It has been traditionally used in folk medicine as a crude drug for jaundice, skin burns and inflammatory, infectious, and hyperlipidemia diseases [1,4,5]. Previous phytochemical investigations showed that the root of *P. cuspidatum* contained a large number of quinones, stilbenes, flavonoids, counmarins, ligans [1,6,7,8]. Moreover, it’s recently reported that the root of *P. cuspidatum* showed significant anti-cancer activity, and the active constituents are anthraquinone and stilbenes, including resveratrol and emodin [9,10,11,12,13,14,15].

**Figure 1:**
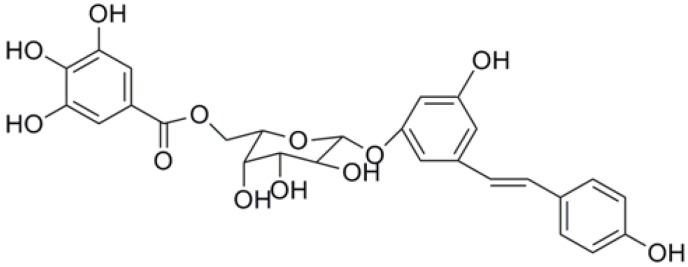
Chemical structure of RESG.

As part of our continuing investigations on natural products with anti-cancer activity, we chose the root of *P. cuspidatum* as our subject of investigation, and a series of compounds were isolated from the root. We found that the resveratrol-4-*O*-d-(2'-galloyl)-glucopyranoside (RESG, Figure 1) had significant anti-cancer effect against hepatocellular carcinoma *in vitro* in our preliminary experiments. There have been no reports regarding the anti-cancer effect of RESG on hepatocellular carcinoma, so the aim of our study is to systematically investigate the anti-cancer activity and mechanisms of action of RESG against hepatocellular carcinoma based on our preliminary experiments, which might provide a scientific basis for the clinical use of RESG.

## 2. Results and Discussion

### 2.1. RESG Has *In Vitro* Antitumor Activity

#### 2.1.1. RESG Inhibited Human Hepatoma Cells Activity

**Figure 2:**
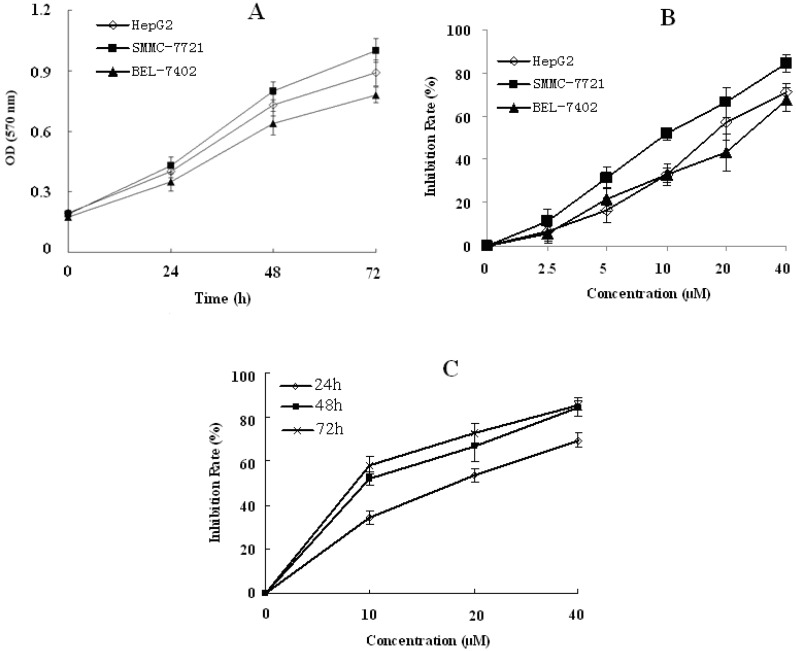
The inhibitory effects of RESG on hepatoma cells. (**A**) The kinetics curves of three human hepatoma cell lines. HepG2, SMMC-7721 and BEL-7402 cells were seeded in 96-well plates, and incubated for 24, 48, 72 h, respectively, then the cell viabilities were determined by an MTT assay; (**B**) HepG2, SMMC-7721 and BEL-7402 cell were treated with 2.5, 5, 10, 20 and 40 μM RESG for 48 h, and the inhibition rate was calculated by an MTT assay; and (**C**) SMMC-7721 cells were treated with 10, 20 and 40μM RESG for 24h, 48 h and 72 h, respectively, then the inhibition rate was calculated by an MTT assay. All results are shown are means ± SD.

Three human hepatoma cell lines (HepG2, SMMC–7721 and BEL-7402) were treated with RESG at concentrations ranging from 2.5–40 μM for 48 h and 72 h, and the rates of inhibition of the three human hepatoma cell types were examined by an MTT assay. RESG showed strong cell growth inhibition effects on all three hepatoma cells in a dose-dependent manner. SMMC-7721 was the most susceptible cell line among the three tested cell lines (Figure 2A,B). RESG at a dose of 40 μM also showed a time-dependent increased inhibition rate, but the inhibition rate was not significantly increased when SMMC-7721 was incubated for 72 h compared to 48 h (Figure 2C). These results demonstrate that RESG significantly inhibits hepatoma cell activity in a dose- and time-dependent manner.

#### 2.1.2. RESG Induced SMMC-7721 Cell Apoptosis

**Figure 3:**
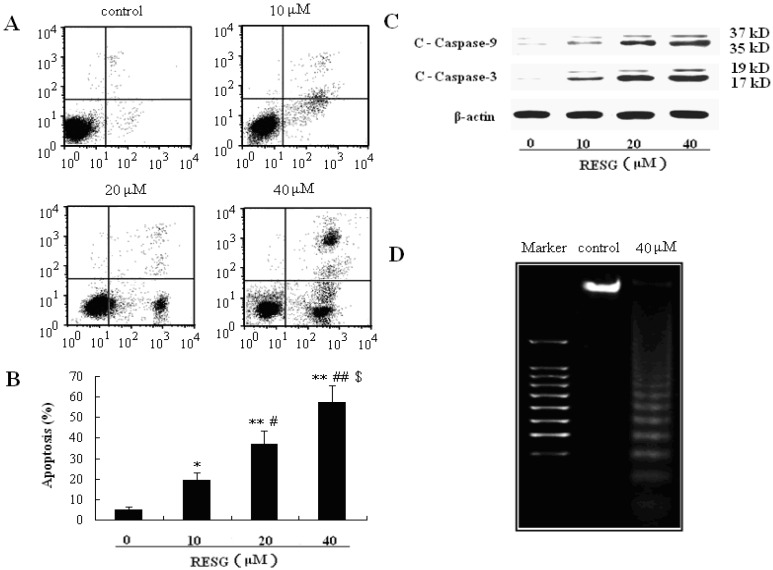
Apoptosis in SMMC-7721 cells. (**A** and **B**) SMMC-7721 cell was treated with 10, 20 and 40 μM RESG for 48 h. Cells were stained with Annexin V/PI, and then the apoptosis was determined using flow cytometry. Data were expressed as means ± SD. * *p* < 0.05, ** *p* <0.01 *vs.* 0 μM RESG; # *p* < 0.05 *,* ## *p* < 0.01 *vs.* 10 μM RESG; $ *p* < 0.05 *vs.* 20 μM RESG; (**C**) SMMC-7721 cells were treated with 10, 20 and 40 μM RESG for 48 h. Total proteins were extracted and detected by western blot analysis using antibodies against C-Caspase-3 and C-Caspase-9; and (**D**) SMMC-7721 cellw were treated with 40 μM RESG for 48 h, and then the DNA fragmentation was determined.

Apoptosis is one of the major mechanisms of cell death in response to cancer therapies, and caspase 3 and caspase 9 are the crucial proteins involved in apoptosis [16]. We studied the effect of RESG on the induction of SMMC-7721 cell apoptosis. Flow cytometry analysis using Annexin V/PI staining revealed that 10, 20 and 40 μM of RESG for 48 h could significantly increase SMMC-7721 cell apoptosis compared to the medium group (Figure 3A,B). To further confirm that RESG could induce SMMC-7721 cell apoptosis, the crucial proteins involved in apoptosis (caspase 3 and caspase 9) were analyzed. The results demonstrated that RESG resulted in cleavage/activation caspase 3 and caspase 9 (Figure 3C). In addition, DNA ladder analysis further confirmed that exposure of SMMC-7721 cells to 40 μM RESG for 48 h could induce an obvious increase in cellular DNA degradation (Figure 3D). All together, our findings reveal that RESG has antitumor effects by induction of apoptosis.

### 2.2. RESG Exhibits Antitumor Effects on SMMC-7721 Xenograft Model Mice

**Figure 4:**
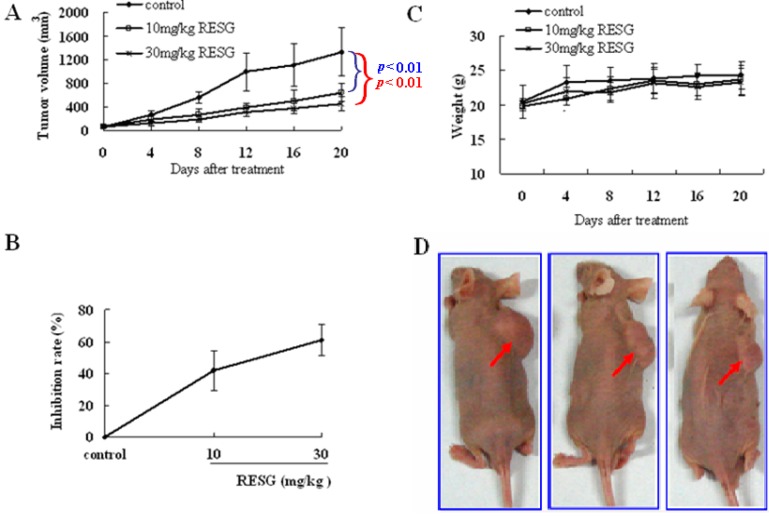
Antitumor effect of RESG in SMMC-7721 xenograft model mice *in vivo*. 4 × 10^5^/0.2 mL SMMC-7721 cell was inoculated into nude mice subcutaneously, and then the mice were randomly divided into control group and two treatment groups (10 mg/kg and 30 mg/kg RESG, respectively). (**A**) Tumor volume curve; (**B**) The inhibition rate of tumor growth was calculated using the formula: Inhibition ratio (%) = [(W_control_ – W_treated_) / W_control_] × 100%; (**C**) Body weight curve; and (**D**) Representative photographs of nude mice bearing SMMC-7721 cell at the end of study.

In order to confirm whether RESG possessed potential antitumor effects on human hepatoma *in vivo*, RESG was evaluated by using SMMC-7721 xenograft model nude mice. Herein, doses of 10 and 30 mg/kg of RESG were applied for 12 days. To quantify the antitumor effects of RESG, the mean inhibition ratio and tumor volume were calculated. The results showed that 10 and 30 mg/kg of RESG could significantly inhibit tumor growth, and the mean tumor volume in nude mice was much smaller than tumors in the control mice (Figure 4A,D). The mean inhibition ratios of tumor growth produced by 10 and 30 mg/kg of RESG were 41.7 ± 12.45% and 60.9 ± 9.9%, respectively (Figure 4B). In addition, there was no significant difference in body weight between the RESG treatment group and the control group (Figure 4C), indicating that no severe toxicity was observed. The above results suggest that RESG at the doses of 10 mg/kg and 30 mg/kg possesses potential antitumor effects against human hepatoma *in vivo*.

### 2.3. Investigation of the Antitumor Mechanisms of RESG *In Vitro*

#### 2.3.1. RESG Affected the MAPK Pathways

**Figure 5:**
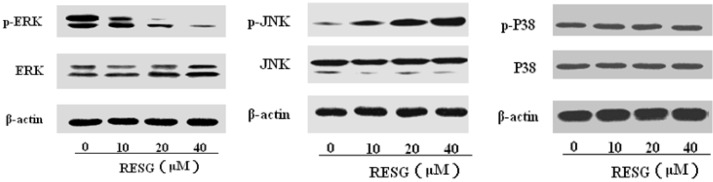
The protein expression of MAPKs. SMMC-7721 cells were treated with 0, 10, 20 and 40μM RESG for 48h, respectively. Total protein were extracted and detected by western blot analysis using antibodies against ERK1/2, p-ERK1/2, JNK, p-JNK, P38 and p-P38.

The above results demonstrate that RESG has significant antitumor effects on human hepatoma *in vitro* and *in vivo*. Next we investigated the mechanism(s) by which RESG induces apoptosis. There have been no reports regarding the antitumor effect of RESG on hepatocellular carcinoma. RESG is one of the stilbene constituents in *P. cuspidatum* like resveratrol, thus we hypothesized that the antitumor mechanisms of RESG might be similar to those of resveratrol. Some studies have reported that the mechanisms of the antitumor effects resveratrol were related to its antiestrogenic activity, activating the expression of p53, Fas-Fas ligand system, protein kinase C, and mitogen-activated protein kinase (MAPK) [17]. The mitogen-activated protein kinase (MAPK) pathways consist of three parallel serine-threonine kinase modules involved in the regulation of diverse cellular events, including proliferation, differentiation and apoptosis. These consist of the c-Jun-N-terminal kinase (JNK), extracellular signal-regulated protein kinase (ERK) and the P38 MAPK [18,19,20]. In most systems, the ERK module plays a cytoprotective role, whereas the JNK and P38 MAPK cascades exert proapoptotic functions [21,22]. Therefore, we speculated that MAPK pathway maybe play a key role in RESG- induced apoptosis to reveal antitumor effects. Then we determined the expressions of JNK, ERK and P38 proteins and phosphorylation proteins expressions implicated in apoptosis regulation. The study revealed that exposure of SMMC-7721 cell to RESG for 48 h resulted in an obvious increase in the p-JNK and decrease in the p-ERK levels. In contrast, RESG had little or no effect on expressions of total protein or p-P38 (Figure 5). These results revealed that RESG could up-regulate the p-JNK expressions which exert proapoptotic functions; however, it could down-regulate the p-ERK expressions which play cytoprotective roles. Thus, JNK and ERK may play critical roles in the RESG-induced antitumor effects.

#### 2.3.2. Activation of JNK and ERK Play Important Roles in RESG-Mediated Apoptosis in SMMC-7721 Cells

**Figure 6:**
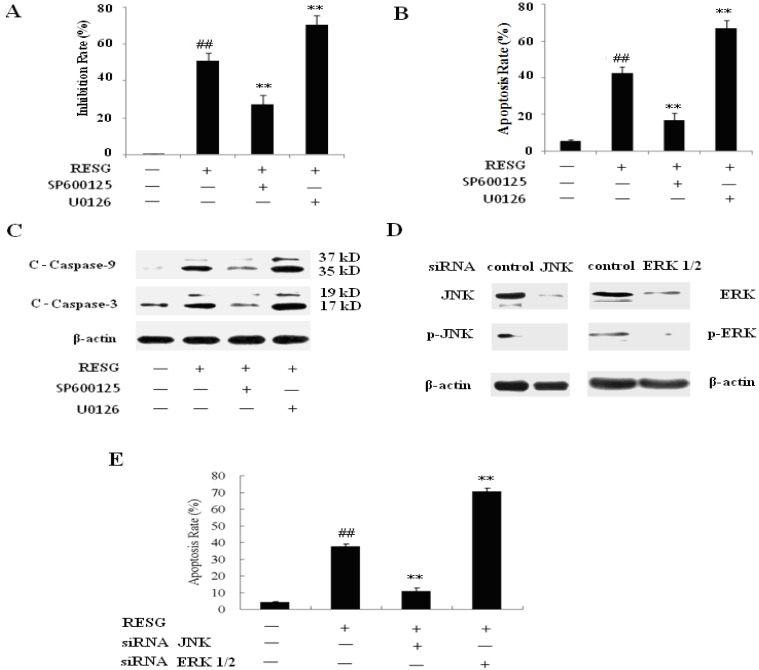
Effects of inhibition JNK and ERK on RESG-mediated antiumor effect. SMMC-7721 cell was pretreated with SP600125 (10 μM) and U0126 (20 μM) for 2 h, and then it treated with 20 μM RESG for 48 h, (**A**) the inhibition rates were calculated by MTT assay. Data were expressed as means ± SD. ** *p* < 0.01 *vs.* RESG; ^##^ *p* < 0.01 *vs.* control; (**B**) the cells were stained with Annexin V/PI, and then the apoptosis was determined by using flow cytometry. Data were expressed as means ± SD. ** *p* < 0.01 *vs.* RESG; ^##^ *p* < 0.01 *vs.* control; (**C**) total proteins were extracted and detected by western blot analysis using antibodies against C-Caspase-3 and C-Caspase-9. SMMC-7721 cell was transfected with ERK1/2 siRNA and JNK siRNA for 48 h, respectively, and then treated with and without 20 μM RESG for an additional 48 h. After treatment, (**D**) the cells were harvested, then total proteins were extracted and detected by western blot analysis using antibodies against ERK1/2 and JNK; (**E**) the cells were stained with Annexin V/PI, and then the apoptosis was determined by using flow cytometry. Data were expressed as means ± SD. ** *p* < 0.01 *vs.* RESG; ^##^ *p* < 0.01 *vs.* control.

To determine whether activation of JNK and ERK play critical roles in RESG-mediated apoptosis in SMMC-7721 cells, these were exposed to RESG in the presence or absence of JNK inhibitor (SP600125, 10 μM) or ERK inhibitor (U0126, 20 μM). Then the cell viability, apoptosis, Caspase-3 and Caspase-9 levels were monitored. The results showed that administration of a nontoxic concentration of SP600125 could increase the viability of SMMC-7721 cells treated with 20 μM RESG (Figure 6A). SP600125 could induce a pronounced decrease in apoptosis (Figure 6B) and significant decreases in cleavage/activation of SMMC-7721 cell’s caspase-3 and caspase-9 (Figure 6C). The results also showed that administration of a nontoxic concentration of U0126 could obviously decrease the viability of SMMC-7721 cells treated with 20 μM RESG (Figure 6A). U0126 could induce a pronounced increase in apoptosis (Figure 6B) and cleavage/activation of caspase 3, caspase 9 of SMMC-7721 cells treated with 20 μM RESG (Figure 6C). Collectively, these results indicate that JNK and ERK play important functional roles in RESG-induced apoptosis.

To further demonstrate that JNK and ERK play important roles in RESG induced apoptosis, we used ERK1/2 siRNA and JNK siRNA to silence ERK1/2 and JNK expression, respectively, then to investigate its impact on RESG-induced apoptosis. Transfection of ERK1/2 siRNA reduced the basal levels of ERK1/2, and transfection of JNK siRNA reduced the basal levels of JNK, indicating the successful inhibition of ERK1/2 and JNK pathway (Figure 6D). Correspondingly, we noted that apoptosis induction by RESG was also increased in cells transfected with ERK1/2 siRNA (Figure 6E) and apoptosis induction by RESG decreased in cells transfected with JNK siRNA (Figure 6E). These results again indicate that ERK and JNK play important functional roles in RESG-induced apoptosis.

## 3. Experimental

### 3.1. Reagents

Methylthiazdyldiphenyltetrazolium bromide (MTT), ERK inhibitor (U0126) and JNK inhibitor (SP600125) were purchased from Sigma (St. Louis, MO, USA). Annexin V-FITC Apoptosis Detection Kits and BeyoECL Plus kits were purchased from Beyotime (Shanghai, China). Caspase 3, Caspase 9, p-JNK, p-ERK, p-P38 monoclonal antibody, JNK siRNA and ERK1/2 siRNA were purchased from Cell Signaling Technology (Beverly, MA, USA). Silica gel was purchased from Qingdao Haiyang Chemical Co., Ltd. (Qingdao, China). Acetonitrile was purchased from the Fisher Chemical (Shanghai) Co., Ltd. (Shanghai, China). All other chemical regents were analytical grade, and purchased from Sinopharm Chemical Reagent Co., Ltd. (Shanghai, China).

### 3.2. Isolation and Preparation of RESG

Dried roots of *P. cuspidatum* were purchased from Tong-Jun-Ge Traditional Chinese Medicine store (Chongqing, China), and the voucher specimen (S1109-HZ) was kept in our laboratory. The comminuted roots were extracted two times with six volumes of 70% aqueous ethanol under reflux (each extraction was lasted 3 h). The combined extract solution was evaporated under vacuum to obtain the crude total extract. Then, the total extract was extracted with ethyl acetate and *n*-butanol, and the *n*-butanol fraction was fractionated by column chromatography using silica gel (200-300 mesh) eluting with chloroform-methanol mixtures of increasing polarity (30:1, 15:1, 10:1, 8:1, 5:1, 2:1). Using TLC analysis, the similar fractions among the sub-fractions of the *n*-butanol fraction were combined, giving seven sub-fractions (Fra. 1–Fra. 7). After that, Fra. 3 was purified by an Agilent 1200 HPLC system (Agilent Technologies, Santa Clara, CA, USA) equipped with a C_18_ (150 × 4.6 mm i.d.; 5 μm) column (Agilent Technologies), The flow rate was 1.0 mL/min, and the solvent was acetonitrile and 0.05% aqueous trifluoroacetic acid (20:80 *v*/*v*). Finally, the solution was freeze-dried after removing the acetonitrile, yielding the RESG which was identified by NMR and compared with the reference literature data [6].

### 3.3. Cells Culture

Human hepatoma cell lines of HepG2, SMMC-7721 and BEL-7402 were purchased from American Type Culture Collection. The cell lines were cultured in RPMI1640 medium (Gibco, Grand Island, NY, USA) supplemented with 10% fetal bovine serum (Gibco) and antibiotics (100 U/mL penicillin and 100 μg/mL streptomycin). The cell lines were keep at a humidified atmosphere of 5% CO_2_ at 37 °C.

### 3.4. Cell Viability and Apoptosis Assay

The cell viability was evaluated using the MTT assay. The inhibition rate was calculated according to the following formula [23]:

Inhibition ratio (%) = (OD_control_ – OD_treatment_) / OD_control_ × 100% (1)

The cells were stained with Annexin V-FITC and PI, and then detected with flow cytometry (FCM) to evaluate apoptosis according to the manufacture’s protocol. DNA ladder was practiced according to Genomic DNA Purification Kit (Promega, Madison, WI, USA), and DNA ladders were visualized by UV illumination after staining with ethidium bromide [24].

### 3.5. Western Blot Analysis

After treatment with RESG, total cells were harvested and homogenized with lysis buffer. The total cellular protein extracts were separated by SDS-polyacrylamide gel electrophoresis, and transferred to nitrocellulose membrane. Membranes were blocked with 5% fat-free dry milk in 1×TBS containing 0.05% Tween 20 under room temperate for 2 h and incubated with primary antibodies at 4 °C overnight. Protein bands were detected by incubation with horseradish peroxidase-conjugated antibodies, and visualized with BeyoECL Plus reagent as reported. Western blot analysis was performed. Protein levels were normalized to β-actin. Fold changes were determined.

### 3.6. Gene Silencing Using Small RNA (siRNA)

Cells were seeded in 24-well plates and transfected with 100 nM JNK siRNA and 100nM REK1/2 siRNA for 48 h, respectively, using the transfection reagent of LipofectamineTM 2000 (Invitrogen, Carlsbad, CA, USA) according to the manufacturer’s protocol. Then gene silencing effects were evaluated by western blot analysis as described above.

### 3.7. Xenograft Assay *In Vivo*

Male athymic pathogen-free nude mice, 4–6 week-old, were obtained from the Experimental Animal Center of the Third Military Medical University, and the experiments were performed in accordance with the National Guidelines for Animal Care and Use. A total of 4 × 10^5^/0.2 mL SMMC-7721 cells were inoculated subcutaneously into nude mice. When the tumors grew to approximate 5–6 mm in diameter, the mice were randomly divided into a control group and two treatment groups (six mice/group). For the two treatment groups, the nude mice were subcutaneously administered RESG at the doses of 10 mg/kg and 30 mg/kg, respectively. The control group was only administered normal saline. Tumor size and body weight were measured each 4-day for 20 days after treatment. The tumor volume (V) was measured and calculated using the formula:

V (mm^3^) = 1/2 ab^2^ (2)

where a and b represent the long diameter and perpendicular short diameter (mm) of the tumor, respectively. In the end of experiments, mice were sacrificed, and the transplanted tumors were taken out and weighed. The inhibition rate of tumor growth was determined and calculated using the formula:

Inhibition ratio (%) = [(W_control_ – W_treated_) / W_control_] × 100% (3)

### 3.8. Statistical Analysis

All results were presented as mean ± standard deviation (SD) from at least three independent experiments. One-way ANOVA was used to test differences for single group analysis, followed by Tukey’s multiple comparisons. Differences with the *p* value less than 0.05 was considered statistically significant.

## 4. Conclusions

In the present study, we observed that RESG significantly inhibited human hepatoma cell viability and induced apoptosis *in vitro*. RESG also showed efficacy *in vivo* in a xenograft model. The study of the molecule’s mechanisms of action showed RESG modified the MAPKs pathway, and RESG increased the p-JNK expression which exerted proapoptotic functions, however, it decreased the p-ERK expression which plays a cytoprotective role. The above results suggest that RESG may be a potential therapeutic agent for hepatocellular carcinoma acting via the JNK and ERK pathways to induce apoptosis. These studies provided a basis for further development of RESG as an anticancer agent.
